# In Silico Analysis of Selected Glycyrrhiza glabra (Licorice) Constituents: Exploring Their Modulatory Effects on Human Superoxide Dismutase, Human Phosphodiesterase-9 and Human Dipeptidyl Peptidase-4

**DOI:** 10.7759/cureus.53494

**Published:** 2024-02-03

**Authors:** Naveena Tamilarasu, Radhakrishnan Narayanaswamy, Vasantha-Srinivasan Prabhakaran

**Affiliations:** 1 Biochemistry, Saveetha Medical College and Hospital, Saveetha Institute of Medical and Technical Sciences, Chennai, IND; 2 Bioinformatics, Saveetha School of Engineering, Saveetha Institute of Medical and Technical Sciences, Chennai, IND

**Keywords:** human dipeptidyl peptidase-4 (hdpp 4), human phosphodiesterase-9 (hpde 9), human superoxide dismutase (hsod), docking, good health and well-being, glycyrrhiza glabra

## Abstract

Background

*Glycyrrhiza glabra* (Licorice) has been known for its various biological activities. In the current investigation, we aimed to evaluate 11 (10 natural and one synthetic) selected constituents of *G. glabra* as potent modulatory agents of human superoxide dismutase (hSOD), human phosphodiesterase-9 (hPDE 9) and human dipeptidyl peptidase-4 (hDPP 4) using *in silico* method.

Methodology

The 11 selected constituents of *G. glabra* (Licorice) were investigated on the docking behaviour of hSOD, hPDE 9 and hDPP 4 by using the PatchDock method. In addition to docking, toxicity analysis was also carried out using the pkCSM free online server (University of Melbourne, Melbourne, AUS).

Results

Toxicity analysis has shown that four ligands (36%) of *G. glabra* (Licorice) are predicted to have human ether-a-go-go-related gene-2 (hERG 2) inhibition activity. The docking analysis showed that glabridin (-224.13 kcal/mol) has shown the highest atomic contact binding energy with the hSOD enzyme, whereas carbenoxolone has shown the maximum atomic contact binding energy with both the hPDE 9 and hDPP 4 enzymes (-239.57 and -173.50 kcal/mol) respectively.

Conclusion

Thus the present finding provides new information about 11 selected ligands of *G. glabra* (Licorice) as potent modulatory agents of hSOD, hPDE 9 and hDPP 4.

## Introduction

The plant genus *Glycyrrhiza* comprises more than 30 species which are commonly found throughout the globe [1]. The *Glycyrrhiza *is the derived from Greek word glykos means “sweet” and rhiza means “root” [2]. Many *Glycyrrhiza *species are cultivated throughout (i) Afghanistan, (ii) China, (iii) France, (iv) Germany, (v) India, (vi) Italy, (vii) Sicily, (viii) Spain, (ix) the UK and (x) the USA. However, *Glycyrrhiza *species have been cultivated in large-scale (commercial) basics in countries like Sicily, Spain and the UK [3]. Among the several *Glycyrrhiza *species, *Glycyrrhiza glabra* (Licorice) is a familiar medicinal herb used in traditional medicine throughout the world owing to its rich ethnobotanical applications to treat several diseases [4]. In India, *G. glabra* (Licorice) has been cultivated in Baramulla, Dehradun, Delhi, Jammu, South India and Srinagar [5]. The rhizomes and roots are well-recognized medicinal parts of *G. glabra* [4]. The roots of *G. glabra* (Licorice) have been used as a flavouring agent in food and candy preparations. Apart from this, roots of *G. glabra* (Licorice) have been used in the preparation of (a) cough syrup, (b) demulcent, (c) mild laxative, (d) expectorant and (e) tonic [5]. The *G. glabra* (Licorice) has been known to possess numerous biological activities such as (i) anti-diabetic, (ii) anti-inflammatory, (iii) anti-oxidant and (iv) hypolipidemic [4,6,7].

Several studies have reported the various phyto-constituents from *Glycyrrhiza* species, especially *G. glabra*, comprise more than 20 tri-terpenoids and approximately 300 flavonoids [8,4]. Moreover, the roots of *G. glabra* (Licorice) have been reported to possess a high content of glycyrrhizin (tri-terpene saponin) [9]. Furthermore, glycyrrhizin and glycyrrhetinic acid are the two major phytoconstituents of the water-soluble fraction, while glabridin is the main constituent of the oil-soluble fraction of *G. glabra* [5]. *G. glabra* (Licorice) has been reported to inhibit acetylcholinesterase (AChE), caspase-3, cyclooxygenase-2 (COX-2), phospholipase A2 and tyrosinase activities [4,10]. The previous reports highlight potential pharmacological activities of crude *G. glabra* (Licorice) extract, which currently motivated us to carry out the present investigation on 10 chosen *G. glabra* (Licorice) constituents which includes 18-beta glycyrrhetinic acid, glycyrrhizic acid, glabridin, liquiritigenin, isoliquiritigenin, glabrene, glyzarin, glabrolide, glabrone, 4’-Methoxy glabridin and one synthetic derivative of glycyrrhetinic acid namely carbenoxolone. These above-mentioned *G. glabra* (Licorice) phyto-constituents were aimed to investigate the docking analysis of human superoxide dismutase (hSOD), human phosphodiesterase-9 (hPDE 9) and human dipeptidyl peptidase-4 (hDPP 4) by employing PatchDock method. These three target enzymes were chosen based on literature research and current research interests to explore their enzyme-modulating activities. In addition, toxicity analysis was also investigated using a pkCSM-free online server (University of Melbourne, Melbourne, AUS).

## Materials and methods

**Figure 1:**
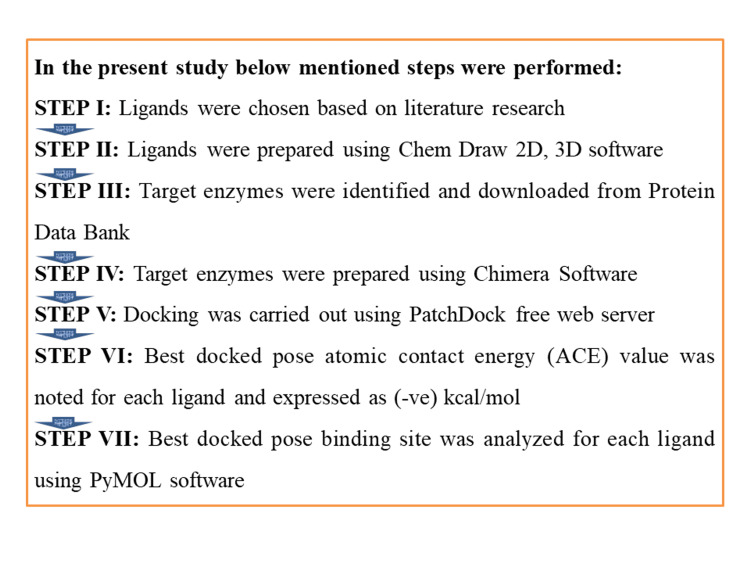
The figure represents the step-by-step docking protocol.

In the present study, *in silico* docking approach was carried out using a free available PatchDock server (Tel Aviv University, Tel Aviv, IL). All 11 ligands of *G. glabra* (Licorice) "simplified molecular input line entry system" (SMILES) were downloaded from the PubChem database. Target enzymes/receptors/proteins were downloaded from the protein data bank (PDB). And moreover, Figure 1 represents the step-by-step docking protocol.

Ligand preparation

Eleven ligands of *G. glabra* (Licorice) were chosen based on the literature search, for instance, glabridin, licochalcone A and isoliquiritin have been known to inhibit tyrosinase enzyme activity. Chemical structures of the 11 *G. glabra* (Licorice) ligands namely (a) 18-beta glycyrrhetinic acid [PubChem ID 44435791]; (b) glycyrrhizic acid [PubChem ID 14982]; (c) glabridin [PubChem ID 124052]; (d) liquiritigenin [PubChem ID 114829]; (e) isoliquiritigenin [PubChem ID 638278]; (f) glabrene [PubChem ID 480774]; (g) glyzarin [PubChem ID 44257206]; (h) glabrolide [PubChem ID 90479675]; (i) glabrone [PubChem ID 5317652]; (j) 4’-Methoxy glabridin [PubChem ID 9927807] and (k) carbenoxolone [PubChem ID 636403] were obtained from PubChem compound database. All 11 (10 natural and one synthetic) ligands were prepared using ChemDraw 2D and 3D (CambridgeSoft, Cambridge, USA) and further subjected to PatchDock [11].

Toxicity analysis

Toxicity analysis was determined for 11 chosen ligands of *G. glabra* (Licorice) using the pkCSM (predicating the small molecule pharmacokinetic (pk) properties using the graph-based signature method) free online server [12]. SMILES of the selected *G. glabra* (Licorice) ligands were used as input files. Then toxicity parameter was chosen by clicking the cursor. The output file was viewed and results were tabulated.

Identification and preparation of target enzymes

The 3D structure of hSOD (PDB ID: 2C9Vwith a resolution of 1.07 Aᵒ); hPDE 9 (PDB ID: 4Y86 with a resolution of 2.01 Aᵒ) and hDPP 4 (PDB ID: 4A5S with a resolution of 1.62 Aᵒ) was retrieved from PDB database. “A” chain of these enzymes was prepared individually by removing other chains, ligands and even crystallographically observed water molecules by applying the UCSF Chimera software tool (Regents, University of California, San Francisco, USA) [13].

Docking study

A docking investigation was carried out for 11 (10 (natural) and one (synthetic)) selected constituents of *G. glabra* (Licorice) using PatchDock (geometry-based molecular docking (MD) algorithm method) free online server. Furthermore, finally, the binding site interaction analysis was done by using the PyMOL software tool (Schrödinger, New York, USA) [11].

## Results

Toxicity analysis has shown that all 11 (10 natural and one synthetic) ligands of *G. glabra* (Licorice) do not exhibit any hERG 1 inhibition activity (as shown in Table 1). On the other hand, four ligands (glabridin, glabrene, glyzarin and glabrone) of *G. glabra* (Licorice) have been predicated to exhibit hERG 2 inhibition activity. Similarly, two ligands (glyzarin and glabrone) of *G. glabra* (Licorice) have been predicated to possess hepatotoxicity activity.

**Table 1:** Toxicity analysis of 11 Glycyrrhiza glabra (Licorice) ligands using the pkCSM free online server. AMESa - AMES toxicity, hERG 1b - Human ether-a-go-go-related gene inhibitor 1, hERG 2c - Human ether-a-go-go-related gene inhibitor 2, HTd - Hepatotoxicity, SSe - Skin sensitisation, TTf - Tetrahymena pyriformis toxicity (log µg/L), MTg - Minnow toxicity (log mM)

Ligand	AT^a^	hERG-1^b^	hERG-2^c^	HT^d^	SS^e^	TT^f^	MT^g^
18-β-Glycyrrhetinic acid	No	No	No	No	No	2.74	1.03
Glycyrrhizic acid	No	No	No	No	No	2.48	5.59
Glabridin	No	No	Yes	No	No	2.52	0.13
Liquiritigenin	No	No	No	No	No	2.37	1.21
Isoliquiritigenin	No	No	No	No	No	2.43	2.08
Glabrene	No	No	Yes	No	No	2.43	1.22
Glyzarin	No	No	Yes	Yes	No	2.31	-0.30
Glabrolide	No	No	No	No	No	2.98	0.76
Glabrone	No	No	Yes	Yes	No	2.58	-0.01
4’-Methoxy glabridin	No	No	No	No	No	2.54	-0.27
Carbenoxolone	No	No	No	No	No	2.43	-1.47

The docking analysis showed that glabridin has demonstrated the highest atomic contact energy (ACE) (-224.13 kcal/mol) with the hSOD enzyme. In contrast, isoliquiritigenin has exhibited the lowest ACE (-93.98 kcal/mol) with the hSOD enzyme (as shown in Table 2). Four ligands (glycyrrhizic acid, glabridin, liquiritigenin and carbenoxolone) have interacted with the Asn86 amino acid residue of the hSOD enzyme (as shown in Table 2).

**Table 2:** The atomic contact energy (ACE) analysis of 11 Glycyrrhiza glabra (Licorice) ligands with the human superoxide dismutase (hSOD) enzyme using PatchDock. ACE* - Atomic contact energy

Ligand	ACE^*^ (-kcal/mol)	Interaction amino acid residue	Bond distance (Å)
18-β-Glycyrrhetinic acid	189.27	Asp109	3.2
Glycyrrhizic acid	199.82	Arg79; Asn86; Ser102	2.8; 3.2; 3.1
Glabridin	224.13	Asn86	3.0 and 3.2
Liquiritigenin	94.42	Asn86	3.0
Isoliquiritigenin	93.98	No interactions	-
Glabrene	168.98	Thr88	3.3
Glyzarin	169.33	No interactions	-
Glabrolide	115.10	No interactions	-
Glabrone	197.86	Thr88	3.3
4’-Methoxy glabridin	177.02	Arg143	3.3
Carbenoxolone	173.53	Asn86	3.5

Similarly, the docking analysis showed that carbenoxolone (a synthetic derivative of glycyrrhetinic acid) has demonstrated the maximum ACE (-239.57 kcal/mol) with the hPDE 9 enzyme. In contrast, isoliquiritigenin has exhibited the least ACE (-96.23 kcal/mol) with the hPDE 9 enzyme (as shown in Table 3). Four ligands (glycyrrhizic acid, glabridin, liquiritigenin and isoliquiritin) have interacted with the Thr363 amino acid residue of the hPDE 9 enzyme (as shown in Table 3).

**Table 3:** The atomic contact energy (ACE) analysis of 11 Glycyrrhiza glabra (Licorice) ligands with the human phosphodiesterase-9 (hPDE 9) enzyme using PatchDock. ACE* - Atomic contact energy

Ligand	ACE^*^ (-kcal/mol)	Interaction amino acid residue	Bond distance (Å)
18-β-Glycyrrhetinic acid	143.36	His296; Ala452	3.2; 2.8
Glycyrrhizic acid	174.94	Thr302; Glu322; Thr363	2.9 and 3.1; 2.3; 3.2
Glabridin	131.96	Thr363	2.3
Liquiritigenin	132.97	Thr363	2.1
Isoliquiritigenin	96.23	His252; Thr363; Asp402; Tyr424; Gln453	3.1; 3.2; 1.9; 2.3; 3.4
Glabrene	105.50	Glu322	1.5
Glyzarin	112.49	Tyr424	2.7
Glabrolide	178.20	Met365; Ala452	3.4; 2.6
Glabrone	143.26	Asp402; Gln453	2.1; 3.1
4’-Methoxy glabridin	146.13	Tyr424; Gln453	3.0; 3.0
Carbenoxolone	239.57	His252; His296	2.5; 2.6

The docking analysis showed that carbenoxolone (a synthetic derivative of glycyrrhetinic acid) has demonstrated the highest ACE (-173.50 kcal/mol) with the hDPP 4 enzyme. In contrast, liquiritigenin has exhibited the lowest ACE (-4.28 kcal/mol) with the hDPP 4 enzyme (as shown in Table 4). Two ligands (glabridin and liquiritigenin) have interacted with the Tyr195 amino acid residue of the hDPP 4 enzyme (as shown in Table 4).

**Table 4:** The atomic contact energy (ACE) analysis of 11 Glycyrrhiza glabra (Licorice) ligands with the human dipeptidyl peptidase-4 (hDPP 4) enzyme using PatchDock. ACE* - Atomic contact energy

Ligand	ACE^*^ (-kcal/mol)	Interaction amino acid residue	Bond distance (Å)
18-β-Glycyrrhetinic acid	92.54	Arg358; Tyr547; Tyr662	2.8; 3.1; 2.6
Glycyrrhizic acid	94.12	Gln123; Gln153; Tyr238; Ser242; Lys250	3.4; 3.2; 3.3; 2.0 and 2.4; 2.5
Glabridin	43.21	Tyr195; Tyr211	3.0; 3.0
Liquiritigenin	4.28	Tyr195	3.1
Isoliquiritigenin	76.79	Arg358; Tyr662	2.9; 2.3 and 2.6
Glabrene	29.15	No interactions	-
Glyzarin	62.52	Asn151; Gln153	2.8; 3.4
Glabrolide	97.09	Ser209; Arg358; Tyr547; His740	2.3; 3.1; 2.1; 2.6
Glabrone	111.68	Thr522; Lys523	2.6; 3.1
4’-Methoxy glabridin	110.24	No interactions	-
Carbenoxolone	173.50	No interactions	-

## Discussion

The phytoconstituents from *G. glabra* (Licorice) have been reported by adopting ultra-performance convergence chromatography [14], high-performance liquid chromatography [15] and ultra-high performance liquid chromatography along with mass spectrometry techniques [7]. Moreover, the gas chromatography technique has been used for the chemical profiling of *G. glabra* (leaves and roots) [16,7]. Furthermore, four phytoconstituents (licochalcone A and B, glabrone and echinatin) of *G. inflata* have been analyzed by adopting the proton and carbon nuclear magnetic resonance technique [17].

Glycyrrhizin (tri-terpene saponin) of Licorice has been reported to inhibit AChE activity [17]. Glabridin, licochalcone A and isoliquiritin have been reported to inhibit tyrosinase activity [17]. Glycyrrhizic acid has been reported to inhibit cyclooxygenase activity [17]. 18-beta glycyrrhetinic acid has been reported to inhibit 11-beta hydroxysteroid dehydrogenase activity. Moreover, flavonoids of *G. glabra* (Licorice) possess 100 times more antioxidant activity compared to that of vitamin E [15]. Thus the above-mentioned background engaged us to select the target enzymes for the present study namely hSOD (target enzyme 1), hPDE 9 (target enzyme 2) and hDPP 4 (target enzyme 3) respectively.

In general, prior to docking investigation, it is essential to have knowledge of the toxicity profile of selected *G. glabra* (Licorice) ligands which will pay way to reduce drug development costs as well as prevent drug failure owing to their toxicity nature. Interestingly in the present investigation, two ligands (glyzarin and glabrone) have been predicted to exhibit both hERG 2 inhibition activity and hepatotoxicity properties. All new drug candidates under drug development should be subjected to know the hERG channel (human ether-a-go-go-related gene) effect prior to clinical study. In the present study, two ligands of *G. glabra* (Licorice) have failed to adhere to the International Regulatory guideline (ICH S7B). The present finding was on par with the earlier reports [18,6].

The ACE analysis of the current investigation showed the hSOD enzyme as following order: glabridin (-224.13 kcal/mol), ˂ glycyrrhizic acid (-199.82 kcal/mol), ˂ glabrone (-197.86 kcal/mol), ˂ 18-beta glycyrrhetinic acid (-189.27 kcal/mol), ˂ 4’-Methoxy glabridin (-177.02 kcal/mol), ˂ carbenoxolone (-173.53 kcal/mol), ˂ glyzarin (-169.33 kcal/mol), ˂ glabrene (-168.98 kcal/mol), ˂ glabrolide (-115.10 kcal/mol), ˂ liquiritigenin (-94.42 kcal/mol) and ˂ isoliquiritigenin (-93.98 kcal/mol). In the present investigation, four ligands (glycyrrhizic acid, glabridin, liquiritigenin and carbenoxolone) of *G. glabra* (Licorice) have interacted with the Asn86 amino acid residue of the hSOD enzyme. The current finding was on par with the earlier report, where pyridine-polybenzimidazole 2-unit has exhibited interaction with the Asn86 amino acid residue of the hSOD enzyme [13].

The ACE analysis of the current investigation showed with the hPDE 9 enzyme as following order: carbenoxolone (-239.57 kcal/mol), ˂ glabrolide (-178.20 kcal/mol), ˂ glycyrrhizic acid (-174.94 kcal/mol), ˂ 4’-Methoxy glabridin (-146.13 kcal/mol), ˂ 18-beta glycyrrhetinic acid (-143.36 kcal/mol), ˂ glabrone (-143.26 kcal/mol), ˂ liquiritigenin (-132.97 kcal/mol), ˂ glabridin (-131.96 kcal/mol), ˂ glyzarin (-112.49 kcal/mol), ˂ glabrene (-105.50 kcal/mol) and ˂ isoliquiritigenin (-96.23 kcal/mol). In the current study, four ligands (glycyrrhizic acid, glabridin, liquiritigenin and isoliquiritin) of* G. glabra *(Licorice) have interacted with the Thr363 amino acid residue of hPDE 9 enzyme. Similarly in the present investigation, carbenoxolone has shown interaction with both His 252 and His 296 amino acid residue of the hPDE 9 enzyme, which was on par with the previous report [19].

The ACE analysis of the present investigation showed with the hDPP 4 enzyme as following order: carbenoxolone (-173.50 kcal/mol), ˂ glabrone (-111.68 kcal/mol), ˂ 4’-Methoxy glabridin (-110.24 kcal/mol), ˂ glabrolide (-97.09 kcal/mol), ˂ glycyrrhizic acid (-94.12 kcal/mol), ˂ 18-beta glycyrrhetinic acid (-92.54 kcal/mol), ˂ isoliquiritigenin (-76.79 kcal/mol), ˂ glyzarin (-62.52 kcal/mol), ˂ glabridin (-43.21 kcal/mol) and ˂ liquiritigenin (-4.28 kcal/mol). In the present investigation, two ligands (18-beta glycyrrhetinic acid and glabrolide) have interacted with the Tyr547 amino acid residue of the hDPP 4 enzyme. The current finding was on par with the earlier report [20].

Limitations and future recommendations

The present study findings are based on docking analysis which provides new knowledge about these 11 ligands of *G. glabra* (Licorice) as hSOD, hPDE 9 and hDPP 4 enzyme inhibition activities and moreover, it is considered as preliminary research work. Furthermore, in vitro and in vivo experiments are needed to confirm these 11 ligands of *G. glabra* (Licorice) as good modulating action against hSOD, hPDE 9 and hDPP 4 enzyme activities.

## Conclusions

The current study showed that all 11 ligands of *G. glabra* (Licorice) have docked very effectively with the three target enzymes namely hSOD, hPDE 9 and hDPP 4. Interestingly, isoliquiritigenin has shown the lowest ACE with both the hSOD and hPDE 9 enzymes (-93.98 and -96.23 kcal/mol) respectively. Thus, the results of the current study have shown new information about these 11 ligands of *G. glabra* (Licorice) as potential modulating agent against hSOD, hPDE 9 and hDPP 4 concerning the treatments of reactive oxygen species mediated diseases, central nervous system (CNS) diseases and type 2 diabetes mellitus (DM).
